# Web-Based Delivery of an Effective Church-Based Intervention Program to Promote Cancer Screening (Community-based Health litEracy-focused intervention for breast and cervical Cancer Control) Among Korean Immigrant Women in the United States: Randomized Controlled Trial

**DOI:** 10.2196/66092

**Published:** 2025-08-25

**Authors:** Hae-Ra Han, Yoon-Jae Lee, Deborah Min, Joyline Chepkorir, DaSol Amy Hwang, Steve Chae

**Affiliations:** 1 Johns Hopkins School of Nursing, 525 N. Wolfe St, Baltimore, MD, 21205, United States, 1 410-614-2669, 1 410-502-5481; 2 Johns Hopkins Bloomberg School of Public Health, Baltimore, MD, United States; 3 University of Pennsylvania School of Nursing, Philadelphia, PA, United States; 4 New York University Grossman School of Medicine, New York, NY, United States

**Keywords:** health literacy, digital health, cancer screening, community health workers, Korean Americans

## Abstract

**Background:**

Women with limited English proficiency continue experiencing an unequal cancer burden. Non-White immigrant women present with more advanced breast and cervical cancer than non-Hispanic Whites, attributed to significant cultural barriers as well as low health literacy in attempting to navigate the United States health care system for cancer screening. Community-based Health litEracy-focused intervention for breast and cervical Cancer Control (CHECC-uP) was an in-person, community health worker-led intervention, addressing both cultural and health literacy barriers through health literacy education and follow-up counseling with navigation assistance. The in-person program was tested in a large cluster-randomized trial and yielded high efficacy in promoting mammogram and Papanicolaou test screening in Korean-speaking women. With over 90% of Americans now having online access, the in-person program was adapted to web-based delivery.

**Objective:**

This study aimed to evaluate the feasibility, acceptability, and preliminary efficacy of the web version of the intervention—e-CHECC-uP.

**Methods:**

A randomized pilot trial was conducted. A total of 40 women were enrolled and randomized (20 per arm). The study intervention consisted of web-based health literacy education followed by phone counseling with navigation assistance. Study assessments were done at baseline, 3 months, and 6 months. The study’s primary outcomes were cancer screening behaviors verified by medical record review. Upon completion of final study assessments, intervention participants were invited to join postintervention interviews.

**Results:**

In total, 34 women (intervention: n=15; control: n=19) completed the assessment at 6 months, yielding a retention rate of 85%. The intervention participants were highly satisfied with e-CHECC-uP with a median rating of 8 on a 10-point scale. Between-group differences in screening rates were 34.6%, 47.9%, and 37.5%, respectively, for completion of the mammogram, Papanicolaou test, and both at 6 months.

**Conclusions:**

We achieved a high retention rate and high participant satisfaction. Although the study was not powered for significance testing, the e-CHECC-uP intervention resulted in large group differences across all cancer screening outcomes in the pilot sample. Online technology can help address multiple logistical barriers associated with in-person intervention delivery. Our findings suggest that web-based delivery of CHECC-uP may be used to promote cancer screening among immigrant women with limited English proficiency, as a promising avenue to ultimately reduce health disparities in underserved communities.

## Introduction

Despite significant progress in the early detection of breast and cervical cancer in the United States, certain racial and ethnic minoritized groups—particularly those with limited English proficiency (LEP)—continue to experience an unequal cancer burden, which is strongly associated with underusage of cancer screening. Non-White immigrant women present with more advanced disease than do non-Hispanic Whites [1,2], which is attributed to significant cultural barriers [1-3] as well as low health literacy [2-6] in attempting to navigate the health care system in the United States. Korean Americans are the fifth largest Asian population in the United States [7] and represent one of the most vulnerable groups for late cancer diagnosis due to significant language barriers and limited health literacy [6,8].

Community health worker (CHW)–led interventions have grown rapidly in health care as possible solutions to alleviating health inequalities. CHW*-*led interventions have demonstrated to be efficacious and are a commonly used approach in promoting health outcomes among vulnerable populations at risk for health disparities [9-11]. A systematic review revealed that CHW-led interventions promoting cancer screening resulted in 6%‐33% between-group differences for mammography and 7%‐29% for Papanicolaou screening [11]. Likewise, a randomized clinical trial involving Asian immigrant women showed that a CHW-led health literacy-focused intervention was highly efficacious in promoting breast and cervical cancer screening with a group difference of 46% for mammography and 45% for Papanicolaou screening [12].

eHealth—“the use of information and communication technologies for health” [13]—is an effective health communication and educational approach for preventive and self-care practices [14,15]. Today, it is estimated that over 90% of Americans have online access [16]. According to a recent community survey [17], 66% of Korean immigrant women have access to the Internet and use the internet as the main method to obtain health information; 98% of them have access to a mobile phone. In light of high digital accessibility, the potential of eHealth as an effective health communication and education tool has been demonstrated [15]. Although CHW-led interventions have shown efficacy in promoting cancer screening among underserved populations, there remains a critical gap in scalable, accessible delivery models—particularly digital formats—that can extend the reach and impact of these interventions for immigrant women with LEP. This study directly addresses this gap by adapting a previously successful CHW-led, health literacy-focused cancer screening intervention (CHECC-uP: Community-based Health litEracy-focused intervention for breast and cervical Cancer Control [12]) into a web-based format (e-CHECC-uP), leveraging the high rate of digital access among Korean American immigrant women. The purpose of this study was to evaluate the acceptability and feasibility of e-CHECC-uP. The study also aimed to address preliminary efficacy of the e-CHECC-uP intervention in addressing cancer screening and psychosocial outcomes.

## Methods

### Design and Sample

A pilot randomized trial was conducted to test the feasibility, acceptability, and preliminary efficacy of e-CHECC-uP in 40 Korean American women. Eligibility criteria included: (1) women self-identifying as Korean living in the Baltimore-Washington Metropolitan area, (2) aged 21 to 65 years, and (3) overdue for mammogram (>2 y since last mammogram for women 40 y and older) [18] or Papanicolaou smear screening (>3 y since last Papanicolaou test for women 21 y and older) [19]. Study participants were recruited from Korean churches in the Baltimore-Washington Metropolitan area. Using computer-generated random numbers, enrolled study participants who completed the baseline study survey were randomized into either the intervention or the control arms (n=20 each). This study was conducted in line with CONSORT (Consolidated Standards of Reporting Trials) guidelines (Checklist 1).

### E-CHECC-uP Intervention

The study intervention consisted of web-based health literacy education and monthly phone counseling with navigation assistance over 6 months. The prototype intervention CHECC-uP consisted of health literacy education and follow-up phone counseling, both delivered by trained CHWs, and was successfully tested in Korean immigrant women from ethnic churches [12]. The health literacy education, which was delivered in-person, was designed to help Korean American women to better understand essential medical terminology used in mammogram and Papanicolaou test screening with relevant medical instructions, while increasing familiarity with the navigational processes to get these screenings done. In order to scale up, we transformed the print materials used for in-person health literacy education into a web-based platform. Details of the online module development will be described elsewhere. Briefly, we worked with a community advisory board to develop the web-based platform of the health literacy education component of CHECC-uP. The community advisory board consisted of 7 Korean American women aged 40‐65 years residing in California or Maryland. Over 2 months, the advisory board members assessed module contents for its organization, relevance, and comprehension. Also, they reviewed both audio and visual quality of the modules and consistency and overall design of them. The final version consisted of 9 educational modules with an estimated completion time of approximately 30 to 60 minutes.

### Procedures

After the online modules were finalized, a trained bilingual research team worked with 5 ethnic churches to identify potential study participants. Upon verbal verification of study eligibility, potential participants were given detailed information about the study and an informed consent form. As the participants signed the form and completed the baseline questionnaire, each participant was assigned to one of 2 groups. For the intervention group, trained study staff handed the participants a tablet along with a log-in instruction sheet with individualized ID and password. Once successfully logged in, the participants were guided to touch the “Start” button on the screen when they were ready to begin and stated that the narrator would walk them through the modules. The staff stood by to answer any questions while the intervention participants completed the modules. Upon module completion, intervention participants received a hard copy version of the educational contents covered in the modules, along with detailed instructions to enable them to log in and view the modules at their convenience. Within 2‐3 weeks after completing the modules, intervention women received monthly phone counseling for about 6 months. The purpose of phone counseling was for CHWs to address participant questions or concerns about mammogram and Papanicolaou test screening as well as monitor participant progress toward screening completion, while providing necessary navigation assistance. For both the intervention and control groups, a bilingual research assistant provided an informational brochure in Korean about breast and cervical cancer and mammogram and Papanicolaou smear screening.

Trained bilingual staff who were masked to the group assignment collected data at baseline, 3 months, and 6 months. During the follow-up phase of the study, our team developed Qualtrics surveys for participants who were not available to come to our scheduled in-person data collection visit at each community site. In addition, at 6 months, intervention participants were asked to join postintervention in-person or phone interviews to share their experiences with e-CHECC-uP. The main goal of these interviews was to better understand intervention participants’ experiences so that the study team could identify areas for improvement and enhance future implementations. Example questions included: “Please share what you gained from participating in the e-CHECC-uP program. What was the most valuable part or lesson of the program? What made it more valuable than others?” or “What did you like least about the program? What changes would you suggest to help improve the e-CHECC-uP program?*”* All interviews were recorded and transcribed verbatim.

### Measurement

A study questionnaire was developed to collect sociodemographic and medical characteristics of participants. Primary outcomes were age-appropriate mammogram and Papanicolaou test screening uptake [18,19], which were verified by medical record review. Secondary outcomes included health literacy, social support, cancer screening-related self-efficacy, and quality of life. The questionnaire was translated into Korean and available to participants. All study instruments were used at baseline, 3 months, and 6 months except for social support and quality of life, which were administered at baseline and 6 months. In addition, the 6-month survey included items addressing satisfaction with the e-CHECC-uP intervention (intervention group only).

Health literacy was measured with the Assessment of Health Literacy in Cancer Screening (AHL-C). The AHL-C includes a total of 52 items addressing five dimensions of health literacy in the context of breast and cervical cancer screening: familiarity, comprehension, reading ability, navigation, and numeracy [20]. Based on previous research [21], we chose 3 subscales addressing familiarity, comprehension, and reading ability. Example items are “Please read out loud the listed words below” and “Please find the correct meaning of each word listed below” [20]. The AHL-C has been validated among Korean women in the United States [20] and in Korea [22], and Black women living with HIV in the United States [23] with α coefficients ranging from 0.70 to 0.96.

We used the 8-item Medical Outcomes Study Social Support Survey (MOS-SS) to measure social support. This shorter version was modified from the original 19-item MOS-SS and includes the emotional and instrumental domains of social support on a 5-point Likert-type scale (1=none of the time to 5=all of the time), with higher scores indicating greater perceived social support [24]. An example item is “How often is someone available to take you to the doctor if you need it?” The shorter version had excellent reliability coefficients ranging from 0.88 to 0.92 [24].

Cancer screening-related self-efficacy was measured by the Korean translated Breast and Cervical Cancer Self-Efficacy scale which measures how confident a woman is in carrying out each of the 4 tasks in relation to mammogram and Papanicolaou tests, respectively. Each scale uses a 4-point Likert type item (1=not at all confident to 4=very confident) with higher scores indicating higher confidence in getting cancer screening [25]. The reliability of the Korean version had an α coefficient of 0.92.

We assessed quality of life using the Korean version of the EQ-5D [26]. The EQ-5D is a widely used validated instrument measuring quality of life [27]. It defines health in 5D: mobility, self-care, usual activities, pain or discomfort, and anxiety or depression. Each dimension has 3 response levels (no problems, some problems, and extreme problems) [27]. In the validation study, the Korean version of the EQ-5D had good reliability and validity through various methods including test-retest reliability using intra-class correlations and Kappa statistics along with a priori hypothesis testing [26].

### Analysis

Final analysis included data from 34 women who completed 6 months assessments and whose cancer screening status was confirmed objectively by medical records. We performed descriptive statistics using means, SD, and frequencies to summarize sample characteristics and study variables and to compare the primary outcomes of the completion of a mammogram, a Papanicolaou test, or both at 6 months. We also used chi-square tests and independent samples *t* tests to compare intervention and control groups at baseline. Statistical significance was determined at *P*=.05. For secondary outcomes, effect sizes were calculated by dividing the difference in mean changes (from baseline to 3 mo and from baseline to 6 mo) by the pooled SD at baseline [28]. Finally, for postintervention interview data, we used a thematic analysis [29] to identify common themes related to participants’ experiences with the e-CHECC-uP program. In total, 2 trained research assistants independently coded the interview data to identify themes, which were then reviewed to determine those that were most common and relevant. Any discrepancies in coding were resolved through team consensus.

### Ethical Considerations

This study was approved by the Johns Hopkins Medicine institutional review board (IRB00180383). Every participant provided written informed consent before their participation, at which time they were informed of the study’s aims, procedures, and the benefits and potential risks. The voluntary nature of their participation was underscored, and participants could withdraw from the study at any given time without any negative consequences. Participants were compensated US $20 in cash, each at baseline survey and 3-month follow-up, and US $40 in cash at 6-month follow-up. Those who participated in the postintervention interview were compensated an additional US $30 in cash at the end of the interview.

## Results

### Sample Characteristics

**Figure 1.**
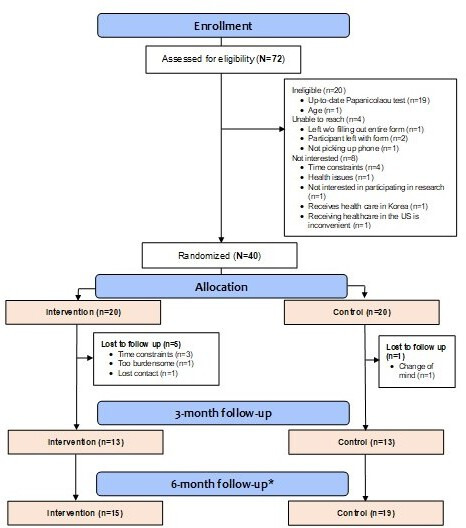
CONSORT (Consolidated Standards of Reporting Trials) flow diagram. The asterisk (*) here indicates that the participants missed the 3-month follow-up but completed the 6-month final survey (n=8).

A total of 52 of the 72 women who were screened for this study were identified as eligible for enrollment. In total, 8 of those eligible declined to participate in our study. In addition, we were unable to reach 4 eligible women after initial contact, yielding a total of 40 women enrolled in the study (77% response rate). A total of 34 participants (intervention: n=15; control: n=19) completed the final study assessment at 6 months (retention rate=85%; refer to Figure 1 for details on recruitment and attrition). Table 1 summarizes participant characteristics at baseline. The final sample was mostly middle-aged (mean 46.3, SD 11.4 years), married (27/34, 79.4%), employed (29/34, 85.3%), and highly educated (years of education: mean 15.6, SD 3.0 years). While more than two-thirds (25/34, 73.5%) reported just okay, difficult, or very difficult to manage with current income, 76.5% (26/34) had limited English proficiency despite living in the United States for an average of 17.3 (SD 10.5) years. The majority of the sample had health insurance (27/34, 79.4%); nevertheless, less than one-third (11/34, 32.4%) indicated that they had a primary care provider at the time of the study. Nearly all (32/34, 94.1%) had access to the internet with 100% (34/34) of the sample reporting mobile phone access. Table 1 also compares the sample characteristics by group. The intervention and control groups were generally compatible at baseline except English proficiency: the intervention group had a significantly higher proportion of women with limited English proficiency (ie, speaking English less than proficient) than the control group (93.3%, 14/15 vs 63.2%, 12/19).

**Table 1.** Sample characteristics at baseline.

Variable	Total (N=34)	Intervention (n=15)	Control (n=19)	*P* value (chi-square or *t* test)
Age (years; range 22‐62 years), mean (SD)	46.3 (11.4)	48.9 (10.1)	44.3 (12.2)	.24
Married, %	79.4	86.7	73.7	.35
Employed full- or part-time, %	85.3	80.0	89.5	.44
Education (years; range 12‐20 years), mean (SD)	15.6 (3.0)	16.0 (0.0)	15.3 (4.2)	.84
Just okay, difficult, or very difficult to manage with current income, %	73.5	80.0	68.4	.45
Speaking English: none, little, or good, %	76.5	93.3	63.2	.04
Years in the United States (years; range 0‐38 years), mean (SD)	17.3 (10.5)	15.7 (11.9)	18.6 (9.2)	.43
Have health insurance, %	79.4	86.7	73.7	.35
Have a primary care provider, %	32.4	40.0	26.3	.40
Have internet access, %	94.1	93.3	94.7	.86
Have mobile phone access, %	100	100	100	—

### Outcome Changes

Table 2 compares the study’s primary outcomes of cancer screening behaviors between intervention and control groups at 6 months. Of 34 women included in the analysis sample, 26, 22, and 14 were overdue for a mammogram, Papanicolaou test, or both tests, respectively, at the time of study enrollment. There were noticeable differences in screening rates between intervention and control groups for completion of mammogram, Papanicolaou test, and both tests at 6 months (34.6%, 47.9%, and 37.5%, respectively).

**Table 2.** Comparison of behavioral outcomes between groups at 6 months (N=34).

Screening test	Intervention group, n/n (%)	Control group, n/n (%)	Difference between groups, %
Mammogram (n=26)	5/12 (41.7)	1/14 (7.1)	34.6
Papanicolaou test (n=22)	5/9 (55.6)	1/13 (7.7)	47.9
Both tests (n=14)	3/6 (50.0)	1/8 (12.5)	37.5

Table 3 presents mean scores of psychosocial outcomes at baseline, 3 months, and 6 months for the intervention and control groups, along with mean change scores and effect sizes. e-CHECC-uP resulted in small to large effect sizes across various psychosocial outcomes measured. Specifically, e-CHECC-uP yielded small effect sizes for reading ability and for both self-efficacy measures related to breast and cervical cancer screening (effect sizes=0.10, 0.06, and 0.06, respectively). One of the dimensions of health literacy—comprehension—resulted in an effect size of 0.27 at 3 months, but the effect was attenuated and in a negative direction at 6 months. Social support was another variable with a medium-size effect of 0.37. Finally, e-CHECC-uP was associated with medium to large effect sizes for a health literacy domain—familiarity—and quality of life with values for each being 0.58 and 0.65 at 6 months.

**Table 3.** Changes in psychosocial outcomes over 6 months (N=34).

Outcome	Values, mean (SD)									Mean change (SD) at 3 months^a^		Mean change (SD) at 6 months^b^			Effect size at 3 months^c^	Effect size at 6 months^d^
	Baseline			3 months			6 months									
	Intervention	Control		Intervention	Control		Intervention	Control		Intervention	Control	Intervention		Control		
Familiarity	22.0 (12.3)	28.9 (13.1)		26.4 (13.5)	33.2 (16.5)		31.1 (11.7)	31.1 (14.6)		3.1 (7.1)	1.7 (8.2)	9.1 (9.6)		1.6 (7.1)	0.11	0.58
Comprehension	5.7 (3.6)	4.4 (3.7)		7.6 (3.6)	5.6 (4.4)		7.7 (4.0)	7.3 (3.8)		2.2 (3.5)	1.2 (3.7)	2.1 (4.5)		2.9 (4.0)	0.27	−0.22
Reading ability	6.6 (3.2)	6.1 (4.2)		7.7 (2.4)	9.2 (3.1)		9.4 (2.1)	9.0 (3.1)		0.7 (2.9)	0.7 (4.6)	2.8 (2.4)		2.4 (3.8)	0	0.1
MOS-SS^e^^,^^f^	27.5 (6.0)	30.4 (7.1)		—^g^	—		29.8 (7.3)	30.2 (6.5)		—	—	2.3 (8.0)		−0.2 (3.7)	—	0.37
Self-Efficacy-B^h^	10.7 (1.4)	11.1 (1.9)		10.5 (1.7)	11.1 (2.1)		10.7 (1.7)	11.0 (1.8)		−0.1 (1.8)	0.0 (1.3)	0.0 (1.8)		−0.1 (1.6)	−0.06	0.06
Self-Efficacy-C^i^	10.5 (1.5)	11.2 (1.6)		10.4 (1.9)	11.3 (2.1)		10.7 (1.4)	11.3 (1.8)		0.1 (2.2)	0.1 (1.8)	0.3 (1.5)		0.2 (2.1)	0	0.06
EQ-5D^e^	8.7 (1.8)	9.7 (0.7)		—	—		7.9 (2.0)	8.1 (2.2)		—	—	−0.8 (2.0)		−1.7 (2.3)	—	0.65

a Mean change from baseline to 3 months.

b Mean change from baseline to 6 months.

c Difference in mean change from baseline to 3 months divided by the pooled SD at baseline.

d Difference in mean change from baseline to 6 months divided by the pooled SD at baseline.

e Assessment performed at baseline and 6 months only.

f MOS-SS: Medical Outcomes Survey-Social Support.

g Not applicable.

h Self-Efficacy-B: Self-Efficacy-Breast Cancer.

i Self-Efficacy-C: Self-Efficacy-Cervical Cancer.

### Acceptability and Feasibility

e-CHECC-uP was well received by the intervention participants. When asked about how satisfied they were with the e-CHECC-uP overall, 11 of 15 (73.3%) rated 7 or higher on a 10-point scale (range 5‐10, median 8, mean 7.6, SD 1.8). In total, 13 (86.7%) women were somewhat satisfied or satisfied with the information they learned about mammogram and Papanicolaou test screening, as well as with the way they learned about the screening; 2 women were not satisfied with either. Similarly, 13 (86.7%) women were somewhat satisfied or satisfied with phone counseling, while 2 women were not satisfied. When given an opportunity to comment further, the 2 participants who were not satisfied said the program was either “too tiresome” or “too difficult for a person who is not familiar or comfortable with English.”

Using postintervention interview data, we identified a few themes addressing the experiences of intervention participants: Recognizing the importance of cancer screening, improved health literacy and self-confidence, and improved knowledge about screening procedures. In terms of recognizing the importance of cancer screening, one participant noted:

*Personally, I knew that screening for breast and cervical cancer is important, but I would always skip it because I was too busy. Through this program, I was convinced and learned that I should actively get the screenings.*

Similarly, another intervention participant shared how e-CHECC-uP helped improve health literacy which led to boosting her self-confidence:

*I am always hesitant to go to the hospital because of the language barrier like the words I am not familiar with, but I gained some confidence through this program.*

Finally, intervention participants talked about how their knowledge about cancer screening procedures improved as a result of their participation in the intervention program. One woman said:

*It was great that I learned about the [cancer] screening procedures and processes through the listening components [of the web modules].*

The trial was feasible in terms of recruitment, retention, and intervention delivery. Specifically, we were able to meet the recruitment goal of 40 women with a response rate of 77%, after accounting for 12 women out of 52 eligible women who either declined (n=8) or were unreachable (n=4). We achieved a retention rate of 85% at 6 months, after accounting for 6 women who were lost to follow-up due to time restraints, participation burden, or loss of contact (Figure 1). Finally, all of our intervention components, including online modules and phone follow-up, were delivered as planned.

## Discussion

### Principal Findings

e-CHECC-uP was highly acceptable and feasible with promising outcome changes. We were able to achieve 85% retention at 6 months with overall high satisfaction ratings in this pilot sample. Given the target population being underserved with limited English proficiency, low health literacy, and limited access to care [6,8], high feasibility and acceptability observed in the pilot sample is encouraging and suggests possible avenues for promising intervention programs to promote breast and cervical cancer screening in Korean immigrant women who often suffer from late diagnoses [1].

Our successful recruitment, retention, and intervention delivery are a result of multiple factors such as authentic care, community partnership, self-directed web-based intervention with human support, and diverse yet standardized modes of communication with study participants [30]. We recruited participants and collected baseline data mainly through face-to-face interactions. As noted by Felsen et al [31], adopting in-person methods may likely have led to successful recruitment as it helps recruiters build rapport with potential study participants. Throughout the study period, we provided various but streamlined approaches for communication. At the earlier stage of the study, phone calls were used as the main method of contact and communication with participants. However, we quickly learned that adding text messaging and email yielded higher rates of response from study participants. Indeed, nearly every woman in the study sample had internet and mobile phone access (Table 1). Additionally, in this sample, 85% participants used texting daily [30], which made it easier to adopt text messaging as one of the main methods of communication. The high rate of internet and mobile phone use among Korean immigrant women with LEP demonstrates strong digital readiness and supports the feasibility of scaling e-CHECC-uP to broader immigrant populations with similar technology access profiles. Taking into account the patterns of technology access and use among the target sample would also facilitate the process of recruitment, retention, and data collection.

e-CHECC-uP pilot resulted in promising group differences across all cancer screening outcomes (range 34.6%-47.9%). While traditional face-to-face CHW interventions designed to promote same cancer screening outcomes have resulted in about 6%‐33% differences [11], the in-person version of this health literacy-focused CHW-led intervention achieved a larger group difference of 46% for mammography and 45% for Papanicolaou test screening [12]. The results indicate that the health literacy-focused approach is effective and perhaps better than the traditional cancer knowledge education. e-CHECC-uP produced comparable preliminary cancer screening outcome changes relative to the original in-person version [12]. This suggests digital CHW-led interventions may serve as scalable alternatives to in-person programs. Additionally, our finding highlights the importance of multilingual digital platforms for delivering health education. Health agencies and payers should invest in scalable, culturally competent eHealth infrastructure to reduce screening disparities. A sufficiently powered, full-scale clinical trial is warranted to rigorously investigate the efficacy of e-CHECC-uP in addressing breast and cancer screening outcomes.

We observed medium to large effect sizes for some of the key psychosocial outcomes including familiarity, social support, and quality of life, whereas self-efficacy showed smaller effects. Additionally, the effect size for comprehension attenuated between the 3- and 6-month follow-up assessments. The reasons for the smaller effect size in self-efficacy and the attenuation in comprehension over time are not entirely clear. One possible explanation may be an artifact of the pilot sample, which included a higher proportion of women with LEP in the intervention arm compared to the control arm (Table 1). The comprehension domain of health literacy required participants to associate each term on the list with a corresponding word or picture, assessing their understanding of the meaning [20]. The reduction in effect size over time may also reflect memory decline related to the learned terms, suggesting a need for reinforcement of health literacy education and an opportunity for scalability through various strategies such as digital refreshers or SMS nudges to sustain health literacy gains during the follow-up period. Existing research documents that people with low health literacy have poorer health outcomes due to its impact on self-efficacy, disease knowledge, or usage of health services [32,33]. For example, diabetic patients with higher health literacy had better understanding of their conditions and self-care behaviors [34,35]. Similarly, other studies support the positive relationship between health literacy and cancer screening, in that people with better health literacy are likely to have a better understanding of cancer screening and improve care-seeking behavior, thereby increasing the use of cancer screening services [36-38]. Postintervention qualitative interviews revealed that e-CHECC-uP helped intervention participants recognize the importance of cancer screening, improved health literacy and self-confidence, and enhanced their knowledge about screening procedures. Future research should investigate the mechanism of e-CHECC-uP for its impact on behavioral outcomes through mediation analyses.

### Limitations

Our study has a few limitations to note. First, due to the nature of a pilot study, there is a small sample size and insufficient power. Additionally, the limited number of intervention participants constrained our ability to systematically integrate qualitative themes with corresponding quantitative findings. A fully powered trial is warranted to test the efficacy of the e-CHECC-uP intervention program and to enable a more robust integration of quantitative outcomes with participants’ qualitative feedback. In addition, the study sample was recruited from ethnic churches in the east coast region of the United States; hence, the study findings may not be generalizable to women who live elsewhere or who do not attend church. We also note that intervention participants were provided tablet devices on-site with technological assistance from the study team as a part of the web-based health literacy education. Such aspects of the study may also prevent findings from being generalizable to those who may face barriers related to use of and access to technology and the internet [17].

### Conclusions

Online technology allows and engages experiences for learners at relatively low cost, given that facilities and instructors are not required. While prior studies have consistently documented inadequate cancer screening in women with LEP [1,2], few have focused on methods to actually address far-reaching, modifiable barriers such as low health literacy and to improve screening participation. Our use of eHealth to deliver health literacy education may represent a promising strategy for promoting engagement and integrating health literacy skills training aimed at educating and empowering women with LEP to improve their mammogram and Papanicolaou test screening.

## Supplementary material

10.2196/66092 Checklist 1 CONSORT-EHEALTH checklist (V 1.6.1).

## Abbreviations

AHL-C: Assessment of Health Literacy in Cancer Screening
CHECC-uP: Community-based Health litEracy-focused intervention for breast and cervical Cancer Control
CHW: community health worker
CONSORT: Consolidated Standards of Reporting Trials
e-CHECC-uP: web-version of Community-based Health litEracy-focused intervention for breast and cervical Cancer Control
LEP: limited English proficiency
MOS-SS: Medical Outcomes Study Social Support Survey
